# Increasing Iron and Zinc in Pre-Menopausal Women and Its Effects on Mood and Cognition: A Systematic Review

**DOI:** 10.3390/nu6115117

**Published:** 2014-11-14

**Authors:** Karla A. Lomagno, Feifei Hu, Lynn J. Riddell, Alison O. Booth, Ewa A. Szymlek-Gay, Caryl A. Nowson, Linda K. Byrne

**Affiliations:** 1 School of Psychology, Deakin University, 221 Burwood Highway, Burwood, Melbourne, Victoria 3125, Australia; E-Mail: klomagno@deakin.edu.au; 2 Centre for Physical Activity and Nutrition Research, School of Exercise and Nutrition Sciences, Deakin University, 221 Burwood Highway, Burwood, Melbourne, Victoria 3125, Australia; E-Mails: feifeih@deakin.edu.au (F.H.); lynn.riddell@deakin.edu.au (L.J.R.); alison.booth@deakin.edu.au (A.O.B.); ewa.szymlekgay@deakin.edu.au (E.A.S.-G.); caryl.nowson@deakin.edu.au (C.A.N.)

**Keywords:** iron deficiency, zinc deficiency, pre-menopausal women, depression, cognition

## Abstract

Iron and zinc are essential minerals often present in similar food sources. In addition to the adverse effects of frank iron and zinc-deficient states, iron insufficiency has been associated with impairments in mood and cognition. This paper reviews current literature on iron or zinc supplementation and its impact on mood or cognition in pre-menopausal women. Searches included MEDLINE complete, Excerpta Medica Database (EMBASE), psychINFO, psychARTICLES, pubMED, ProQuest Health and Medical Complete Academic Search complete, Scopus and ScienceDirect. Ten randomized controlled trials and one non-randomized controlled trial were found to meet the inclusion criteria. Seven studies found improvements in aspects of mood and cognition after iron supplementation. Iron supplementation appeared to improve memory and intellectual ability in participants aged between 12 and 55 years in seven studies, regardless of whether the participant was initially iron insufficient or iron-deficient with anaemia. The review also found three controlled studies providing evidence to suggest a role for zinc supplementation as a treatment for depressive symptoms, as both an adjunct to traditional antidepressant therapy for individuals with a diagnosis of major depressive disorder and as a therapy in its own right in pre-menopausal women with zinc deficiency. Overall, the current literature indicates a positive effect of improving zinc status on enhanced cognitive and emotional functioning. However, further study involving well-designed randomized controlled trials is needed to identify the impact of improving iron and zinc status on mood and cognition.

## 1. Introduction

Iron insufficiency, or mild iron deficiency (ID), in the absence of anaemia is defined as depleted iron stores. When the absence of iron stores affects erythropoiesis, this reduces haemoglobin (Hb) concentration and iron deficiency anaemia (IDA) occurs [1]. ID is the most prevalent single nutrient deficiency in the world, affecting not only those in economically developing countries, but also individuals in developed countries [2,3]. Pre-menopausal women are at greater risk of ID due to their regular menstrual blood loss and elevated iron requirements in pregnancy [4,5]. Iron and zinc are often present in similar dietary sources, such as whole-grain cereals, red meats, legumes, seeds and nuts, as well as seafood, and deficiencies in iron and zinc often co-occur [6,7,8]. However, data on the prevalence of zinc deficiency are lacking, due to the absence of a biochemical marker of total body zinc status. Plasma or serum zinc concentrations, the most widely used indicators of zinc status, are maintained within a narrow range (12–15 μmol/L) and require fasting blood samples for a meaningful test [7,9]. Hair zinc concentration is often used in combination with plasma zinc to provide a good indication of zinc status [10,11]. However, hair zinc alone is better for representing longer-term zinc status. Further, the use of hair zinc concentration as an index of zinc status suffers from limited reference data and variations due to age, sex, season, hair colour and hair growth rate [12]. Thus, alternative methods have been used to estimate zinc deficiency in populations based on reported dietary intake. For example, World Health Organization (WHO) [13] used a factorial approach to estimate the average basal and standard zinc requirements (*i.e.*, minimum amount of zinc to cover losses in individuals adapted or not adapted to low usual dietary intakes of zinc) for population subgroups and then considered issues of zinc bioavailability from the typical diet in various regions of the world to derive estimates of the minimal dietary intake of zinc that would meet these requirements. Using this approach, WHO [14] estimates that zinc deficiency affects around one-third of the world’s population.

Research suggests that ID is strongly linked to cognition, mood, zinc insufficiency and depression. However, the evidence of a causal link though intervention studies is still limited or the results have been mixed. Furthermore, there is limited research assessing the effect of increasing the dietary intakes of both iron and zinc on markers of mood or cognition. This systematic literature review considers randomized controlled trials, investigating the effect of increasing either iron or zinc (either though supplementation or dietary methods) on markers of mood or cognition, primarily in pre-menopausal women.

## 2. Method

### 2.1. Selection Criteria

#### 2.1.1. Participants

Participants were required to be adolescent girls and pre-menopausal women between the ages of 12 and 55 years. Participants were also required to exhibit evidence of either ID or IDA based on Hb concentration, Hb plus ferritin or other author-defined evidence of ID or IDA. No restrictions on participant levels of zinc deficiency were applied to studies evaluating increased zinc intake; however, participants were still required to be females aged between 12 and 55 years. Included studies were required to have a sample size larger than 10. Studies with a population younger than 12, or older than 55 years of age, or that included non-human subjects were also excluded. Studies that included some male participants were only included if the results for females were reported separately.

#### 2.1.2. Types of Studies

Included studies were intervention, placebo-controlled trials with a control group assessing the effect of increasing iron or zinc on markers of mood or cognition. Studies were excluded if they investigated an unrelated disorder (e.g., iron/cognition/Alzheimer’s disease). The intervention period had to be at least 4 weeks in length, and the effects of the intervention on mood or cognition had to be assessed by a recognized assessment tool.

#### 2.1.3. Outcome Measures

Domains of cognitive functioning considered relevant for this review included attention, memory, learning, psychomotor skills and concentration. The mood domain of predominate focus in this review was depression, but also included anxiety, irritability and anger.

### 2.2. Identification of Studies

The original search was conducted in May, 2013 (Appendix 1). Originally, the focus of the review planned to include studies that used dietary methods to increase iron or zinc. However, due to a paucity of these types of studies, the search terms were modified to capture both dietary intake and oral iron supplementation of iron and zinc and to limit research to female participant groups (Appendix 2). Electronic searching was conducted using the following databases: ProQuest Health and Medical Complete, MEDLINE complete, Excerpta Medica Database (EMBASE), PubMed, psychINFO, psychARTICLES, Academic Search complete, Scopus and ScienceDirect between May 2013 and August 2013. Key terms (iron) AND (deficien* OR status OR intake OR “dietary intake” OR nutrition*) and (zinc) AND (deficien* OR status OR intake OR “dietary intake” OR nutrition*) were searched in the title, abstract and keywords and key terms (mood OR affect* OR depress*) and (cognit*), (wom?n OR “female”) in the subject. Iron and zinc were searched separately in relation to their effects on mood and cognition. The search was not limited by language, but included the use of a “peer review only” filter. Articles published between January 1995 and May 2013, were included in the search. The references of identified trials and relevant review articles were examined for eligible studies that may have been missed in the original search.

### 2.3. Screening Process

After electronic searching was complete and duplicates removed, titles and keywords were scanned for relevance; articles were retrieved if these included a combination of iron and/or zinc and mood and/or cognition. Abstracts of the retrieved articles were further screened for relevance, removing papers that were reviews or commentaries. Full-text articles were read, and papers not meeting the inclusion criteria were removed.

### 2.4. Data Extraction

The data extracted included the year of publication, randomization procedures, allocation concealment, blinding, setting, characteristics of participants (e.g., age, country and relevant demographics), characteristics of intervention, baseline iron/zinc status data, information regarding attrition/dropout rates and length of clinical trials. Furthermore, the characteristics of the main outcome measures, analytic methodology and relevant results were extracted for evaluation. Data were extracted by the review author and cross-referenced by another author (Linda K. Byrne).

## 3. Results

### 3.1. Search Results

**Figure 1:**
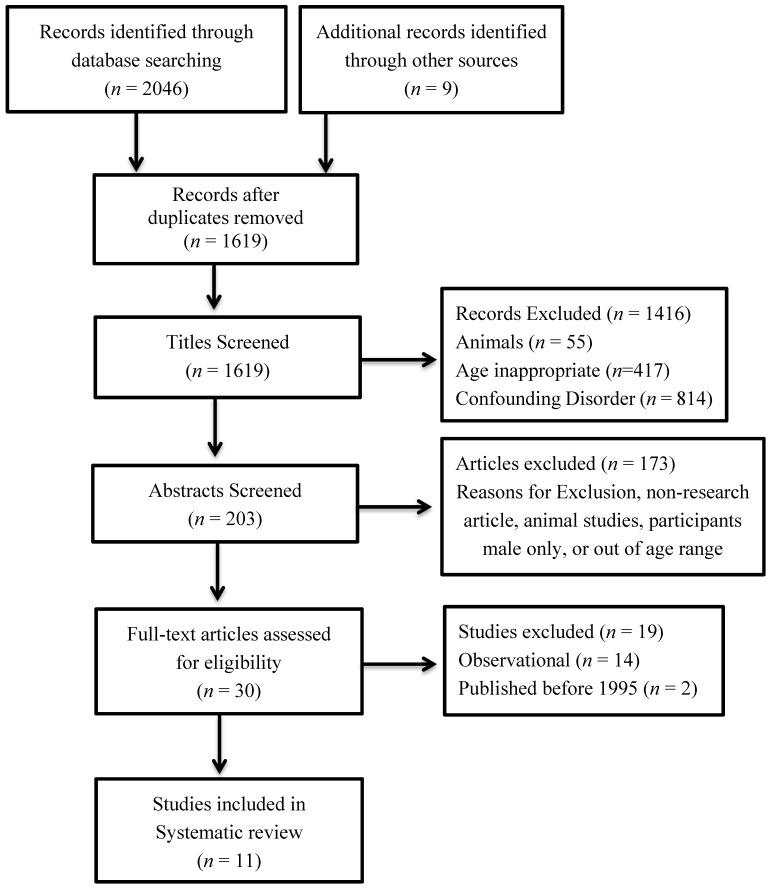
Flow chart of how the literature was selected for inclusion.

A total of 2046 articles were retrieved from database searches, and a further nine were retrieved from additional sources (*i.e.*, reference lists; academic colleagues). The titles and abstracts were screened for eligibility, and 30 full-text articles were assessed for inclusion. Eleven articles met the criteria for inclusion in this review. No articles in a language other than English were retrieved. In total, nine randomized controlled trials and two non-randomized controlled trials were included in the review (Figure 1).

### 3.2. Study Characteristics

A detailed summary of the study characteristics is included in Table 1. Three studies were conducted in the United States [2,15,16]; two were in Poland [17,18], and one study was conducted each in Japan [19], India [20], South Africa [21], France [22], Switzerland [19] and New Zealand [23].

### 3.3. Participant Characteristics

Eight studies included only pre-menopausal women [2,15,16,19,21,22,24]. Three included male participants [17,18,20]. Participants of one study included women who were six weeks postpartum [21], and two studies included participants that met the Diagnostic Statistical Manual of Mental Disorders—Fourth Edition Text Revision (DSM-IV-TR) criteria for major depressive disorder (MDD), differentiating these two populations from those of the rest of the included studies [17,18]. The sample sizes for studies were heterogeneous and ranged from between 20 and 219 participants.

### 3.4. Design

Of the 11 included studies, nine were randomized, placebo-controlled, double-blind procedures [2,15,16,17,19,21,22,23,24]. Of the remaining two studies, one was a non-randomized, placebo-controlled trial and did not explain the blinding procedure [20], and the other did not explicate the allocation process [18]. Three studies looked at the effects of oral iron supplementation on cognition only [2,15,23], while three looked at the effects of oral iron supplementation on just mood [16,22,24]. A further two assessed both variables as outcomes [20,21]. No studies were found that examined the effect of increasing dietary intake of iron and any subsequent effect on mood or cognition*.* The iron status of participants varied between the studies, and Table 1 includes further information regarding the levels used to determine iron status in each individual study. Three studies included ID, IDA treatment and iron-sufficient control groups and examined differences in changes between all three groups [2,16,20]. One included participants with only IDA and excluded those with ID [21]. Four studies included only participants with ID, but failed to have iron-sufficient control groups [15,22,23,24].

Three intervention studies were found looking at the effects of zinc supplementation and depression. One of these looked specifically at the effect of zinc supplementation in depressive symptoms in pre-menopausal women [19]. The two papers remaining, focusing on zinc, looked at the effect of using zinc supplementation as an adjunct to antidepressant therapy. Additionally, both used participants that met the criteria for MDD [17,18]. Similar to the iron studies, no intervention studies were found that assessed the effect of dietary zinc on depressive symptoms. Furthermore, the search yielded no results for intervention trials that fit the inclusion criteria and that specifically examined the effects of zinc supplementation on cognitive functioning.

### 3.5. Measures of Cognition

Outcome measures for cognitive function varied across studies, as did the domain of cognition assessed for each study. Most of the included studies measured multiple domains of cognitive functioning. These included attention, working memory and other aspects of memory (e.g., short-term memory (STM) and long-term memory (LTM)), learning and intellectual abilities. A detailed summary of cognitive outcome measures used in each study is described in Table 2. Three of the 11 included studies measured aspects of attention [2,15,23]. Five assessed aspects of memory [2,15,20,21,23]; three assessed intellectual ability [2,20,21], and two assessed learning [2,21].

### 3.6. Measures of Depression

Outcome measures used to determine depression or depressive symptoms varied considerably between studies. A detailed summary of the outcome assessment tools used for each individual study is outlined in Table 2. Four studies measured depressive symptoms, pre-/post-intervention [17,18,22,24], while a further two assessed general mood state, using subscales of the measures to assess depressive symptoms [16,19,20]. One study used a measure that specifically assessed postpartum depression [21].

### 3.7. Analytic Methodology

Table 2 has a detailed summary of the statistical analyses applied in each study. Eight studies used analysis of variance (ANOVA) [2,15,16,17,18,20,21]; three used multiple linear regression [15,23,24]; one used Pearson’s correlation [16]; and one used a repeated measures analysis of covariance (ANCOVA), with intellectual ability (IQ) as a covariate [2]. One further study used Wilcoxon’s sign-ranked test [19] to assess differences.

**Table 1:** Characteristic summary of the included studies (*N* = 11). ID, iron deficiency.

Study	*n*	Population	Iron or Zinc Intervention	Baseline Iron or Zinc Status	Study Duration and Dropouts	Outcome Measures
Bruner *et al.*, 1996 [15]	81	American adolescent females, ID only, 13–18 years	Daily dose of 1300 mg ferrous sulphate, equivalent to 260 mg elemental iron	**ID** = serum ferritin <12 μg/L, with normal haemoglobin (Hb)	8 weeks 8 dropouts	The Brief Test of Attention, Symbol Digits Modalities. The Visual Search and Attention Test, The Hopkins Verbal Learning Test
				**IDA** ≤ Hb 115 g/L for African American girls or <Hb 120 g/L for caucasian girls		
Beard *et al.*, 2005 [21]	95	South African mothers, 18–30 years	Daily dose of 125 mg ferrous sulphate to anaemic mothers	**IDA** = Hb 90–115 g/L + 2 of the following: mean corpuscular volume <80 femtoliters (fL), transferrin saturation <15%, serum ferritin <12 μg/L	9 months, postpartum 14 dropouts	Edinburgh Post-Natal Depressive Scale, Ravens Coloured Matrices, Perceived Stress Scales
Murray-Kolb and Beard, 2007 [2]	152	American women, 18–35 years	160 mg ferrous sulphate, containing 60 mg elemental iron	**ID** = Hb ≥120 g/L and two or more other abnormal iron values	16 weeks 39 dropouts	Cognitive Abilities Test
				**IDA** = Hb between 105 and 119 g/L and two or more other abnormal iron values		
McClung *et al.*, [16]	219	Female American soldiers, 15–25 years	100 mg ferrous sulphate, 15.0 elemental iron ± 0.2 mg	**ID** ≥ 2 of the following 3 indicators: serum ferritin <12 μg/L, transferrin saturation <16% or red blood cell distribution width >15%	8 weeks 48 dropouts	Profile of Mood States
				**IDA** = ID + Hb of <120 g/L		
Verdon *et al.*, 2003 [24]	144	French females, ID only, 18–55 years	Ferrous sulphate, 80 mg elemental iron as pill	**ID** = not transparent	4 weeks 12 dropouts	Questionnaire bref d’auto-évaluation des dimensions dépressive, asthénique et anxieuse
				**IDA** = Hb <117 g/L		
Vaucher *et al.*, 2012 [22]	198	Swiss females, ID only, 18–50 years	80 mg ferrous sulphate	**ID** = Hb ≥120 g/L, and ferritin <50 μg/L	12 weeks 77 dropouts	Current and Past Psychological Scale
Lambert *et al.*, 2002 [23]	121	Adolescent females from New Zealand, ID only, 12.5–17.9 years	105 mg elemental iron	**ID** = Serum ferritin <12 μg/L, Hb >120 g/L	8 weeks 5 dropouts	Hopkins Verbal Learning Test, Stroop Task Visual Search, Reading Span Task, Visual search task
				**IDA** = Serum ferritin <12 μg/L, Hb <120 g/L		
Devaki *et al.*, 2009 [20]	120	Adolescent male and female students from India, 15–18 years	Iron (III) hydroxide polymaltose complex, 100 mg elemental iron	**ID boys** = Hb ≥115 g/L and transferrin saturation <16%	8 months dropouts not recorded	Ravens Progressive Matrices, Weschler Adult Intelligence Scale, Emotional Quotient
				**ID girls** = Hb ≥105 g/L and transferrin saturation <16%		
				**IDA boys** = Hb <105 g/L and transferrin saturation <16%		
				**IDA girls** = Hb <105 g/L and transferrin saturation <16%		
Siwek *et al.*, 2009 [17]	60	Polish males and females with major depressive disorder, 18–55 years	100–200 mg imipramine (antidepressant) + 25 mg zinc	Serum zinc determined by flame atomic absorption spectrometry; baseline zinc deficiency was unclear	12 weeks 9 dropouts	Hamilton Depression Rating Scale, Beck Depression Inventory, Clinical Global Impression Montgomery—Åsberg Depression Rating Scale
Nowak *et al.*, 2003 [18]	20	Polish males and females with major depressive disorder, 25–57 years	clomipramine 125–150 mg, amitriptyline 125–150 mg, citalopram, 20 mg fluoxetine, 40–20 mg (all standard antidepressant treatment) + 25 mg zinc	Unclear	12 weeks 6 dropouts	Hamilton Depression Rating Scale, Beck Depression Inventory
Sawada and Yokoi, 2010 [19]	60	Japanese females, non-anaemic, 18–21 years	Multivitamins + 7 mg elemental zinc	Serum zinc determined by inductively coupled argon plasma-mass spectrometer; no other details provided on this	10 weeks dropouts not recorded	Profile of Mood States

### 3.8. Evaluation of Validity

Study validity is reported in Table 2. Randomization procedures were outlined in all but two studies [18,20]. The researcher was clearly blinded to intervention in nine studies; the outcome assessor was clearly blinded in four studies, and the participants were clearly blinded in nine studies. Masking was unclear in one study [21] and did not occur in one study [20]. There were dropouts in nine studies [2,15,16,17,18,21,22,23,24]. Reasons for the dropouts were reported for all but one study [19]. An intention-to-treat protocol was used in three studies [15,22,24]. It was unclear how all of the other studies dealt with missing data. Four important areas of bias, including selection, performance, detection and attrition biases, were informally assessed using a bias hierarchy checklist by Wright, Brand, Dunn and Spindler [25].

### 3.9. Results

Table 3 includes a detailed summary of all of the relevant findings for the included studies. Six studies reported increased iron status, as measured by varied markers of iron status in participants treated with iron supplements [2,15,16,20,21,22,23]. It was unclear in one study whether iron status had increased in the iron-treated group [24]. Two zinc studies reported increased serum zinc concentrations, post-treatment with zinc supplementation [17,19]; however, this was unclear in the third zinc study [18].

#### 3.9.1. Iron and Cognition

Despite the heterogeneous nature of the included studies, all that looked at the effect of iron status on cognition found some form of improvement in cognitive functioning after iron supplementation. Of the five studies that assessed the effect of iron supplementation on measures of cognition, two examined the effect of iron status on attention at baseline. Murray-Kolb and Beard [2] reported that an increasing level of severity of ID was associated with decreased cognitive functioning. Specifically, they found that women with ID had a composite baseline score that was significantly worse on tests of attention, learning and memory when compared to iron-sufficient participants. Furthermore, the authors reported that women with IDA performed significantly worse on tasks of attention and memory in comparison to healthy controls. Similarly, Devaki *et al.* [20] reported that adolescents with ID and IDA had lower mean scores on tasks of memory and intellectual ability than healthy controls.

##### Attention and Memory

Of the five studies that assessed the effect of iron supplementation specifically on memory, only one found a significant effect. Murray-Kolb and Beard [2] reported that women who received iron supplementation and were considered serum ferritin responders, that is women who experienced a significant improvement in serum ferritin regardless of whether they initially had ID or IDA, also showed significant improvement on measures of attention. Furthermore, they reported that women who showed a significant improvement in Hb concentration completed tasks significantly faster than those who did not have a significant improvement in Hb. Neither of the studies by Bruner *et al.* [15] and Lambert *et al.* [23] detected significant improvement on measures of attention upon increasing iron status with iron supplementation.

**Table 2:** Validity Characteristics of included studies.

Study	Selection Bias or Detection Bias	Performance or Detection Bias	Attrition Bias	Controlled Variables	Reviewer Notes/Possible Limitations
Bruner *et al.*, 1996 [16]	Randomization clear, double-blinded design	Researchers and participants blinded to allocation	Not all randomized included in outcomes; dropouts and reasons for dropouts reported	Additional vitamins and minerals	All participants had ID. There was no measurement of baseline differences between iron-sufficient and ID adolescents. Serum ferritin was a marker of iron status. Statistical analysis was by group assignment, rather than ferritin response.
Beard *et al.*, 2005 [21]	Randomization clear, double-blinded design	Participants unaware of group allocation	Intention-to-treat protocol utilized to deal with missing data; dropouts reported; however, reasons for dropouts not reported	Age, socioeconomic status, income, education, nutritional intakes	Staff members administered supplementations at health clinics with instructions for the mothers on when to take and when to return, * etc*. Standardized outcome measures were used.
Murray-Kolb and Beard, 2007 [2]	Randomization clear, double-blind placebo-controlled design	One researcher knew the group allocation; outcome assessors, as well as participants were blinded to group allocation	Not all of those randomized included in outcomes; dropout rates reported; however, reasons for dropouts not reported	Socioeconomic status, grade point average, level of physical activity and other demographics;other vitamins/minerals; inflammation, contraception and menstrual cycle; intellectual ability (IQ) was used as a covariate	Standardized and validated measures of were cognition used. High reliability. Analysis was completed on women who were classified as ferritin responders or non-responders. Determination of the relationship between changes in iron status and changes in cognition. 16 weeks used to allow more time for brain iron concentrations to replenish.
McClung *et al.*, 2009 [16]	Randomization reported, double-blind, placebo-controlled design	Researchers, outcome assessors and participants all blinded to allocation	Unclear if all of those randomized included in outcomes; dropout rates and reasons for dropouts reported		Compliance monitored and assessed as a confounding variable. Research assistants administered capsules to participants each day. Treatment occurred on the background of battle combat training. Randomization procedures were not described.
Verdon *et al.*, 2003 [24]	Randomization clear, double-blind design	Researchers, outcome assessors and participants blind to allocation	Intention-to-treat protocol completed to deal with missing data; dropout rates recorded and reasons for dropout recorded	Other disorders that could explain fatigue	Intervention period was relatively shorter than other included studies. A validated self-administered questionnaire was used, although fatigue was assessed on a visual analogue scale. Adherence to treatment was monitored by an electronic device that recorded the date and time that the pill container was opened
Vaucher *et al.*, 2012 [22]	Multi-centre study, randomization clear, double-blind, placebo-controlled design	Researchers, outcome assessors and participants blinded to allocation	Intention-to-treat protocol completed to deal with missing data		A validated self-administered questionnaire used. Intention-to-treat analysis was used, and attrition and compliance were less of an issue. Dropout rates were not reported
Lambert *et al.*, 2002 [23]	Randomization occurred, but allocation procedures not described	Researcher and participants blinded to allocation	Unclear if randomized participants included in outcomes/dropout rates reported/reasons for dropouts reported		Standardized and validated outcome measures were used.
Devaki *et al.*, 2009 [20]	No randomization described	No blinding methods described	Unclear if randomized participants included in outcomes; dropout rates and reasons for dropout reported		Compliance was monitored as supplements were administered by a supervisor at breakfast and lunch. Potential for performance, selection and detection bias.
Siwek *et al.*, 2009 [17]	Randomization clear	Researchers and participants blinded to allocation			Outpatient sample.
Nowak *et al.*, 2003 [18]	Randomization described	Researchers and participants blinded to allocation	Unclear if randomized participants included in outcome; dropout rates reported however reasons for dropout not reported		Clinical sample, so it is difficult to generalize to wider population. Standardized and validated measures of depressive symptoms were used. Allocation procedures were not described.
Sawada and Yokoi, 2010 [19]	Randomization clear, A double-blind, double-blinded, placebo-controlled design	Researchers and participants blinded to allocation	Information around dropout rates and missing data was not reported	Age, height, body mass, body weight	Pilot study, first of its kind. There are missing data and information around how this was dealt with. Treatment was possibly confounded by the use of multivitamins, alongside zinc

**Table 3:** Summary of relevant results. HVLT, Hopkins Verbal Learning Test; MCV, mean corpuscular volume.

1: Iron					
Study	Dependent Variable	Domains	Statistical Analysis	Changes in Iron/Zinc Status	Findings
Bruner *et al.*, 1996 [15]	Cognition	*Attention*: Assessed using Brief Test of Attention (BTA), Symbol Digit Modalities Test (SDMT) and Visual Search and Attention Test (VSAT). *Memory*: multicomponent test, Hopkins Verbal Learning Test (HVLT). *Learning*: multicomponent test, HVLT	Intention-to-treat analysis; multiple linear regression, assessment of post iron treatment cognitive scores; analysis of variance (ANOVA), assessed HVLT learning curve	Iron girls: significantly higher serum ferritin concentration (18.2 (SD 12.6) *vs.* 3.5 (6.6) µg/L, *p* < 0.001) and higher mean Hb concentration (135 (8.0) *vs.* 127 (7.0) g/L, *p* < 0.001)	No significant effect on attention were found. Significant improvement on total recall scores of HVLT for girls who took iron treatment, compared to control group (*p* < 0.02). No significant differences were found between delayed recall and recognition. Learning: All participants recalled more words after each trial (*F* = 273.7), *p* < 0.001), although no significant differences were found between groups. Despite this, participants who took iron treatment performed better compared to the control group (*F* = 6.3, *p* < 0.02).
Beard *et al.*, 2005 [21]	Cognition	*Intellectual abilities*: Raven’s Coloured Progressive Matrices (RPM). *Memory*: Digit Symbol Test	Repeated measures ANOVA; Pearson’s correlation, strength of association between variables	IDA iron group: Significant improvement in Hb transferrin saturation (TSAT) and serum ferritin (Ft) values. IDA placebo: Also significant increase in Hb, reflecting natural state of iron status restoration postpartum. Iron sufficient: No change in haematology	Iron treatment effected significant improvement on RPMs and Digit Symbol scores. Iron-treated mother’s scores were almost identical to non-anaemic mothers at 9 months. An association was found between—RPM scores and Hb, 10 weeks—Digit symbol test and MCV at 9 months
	Emotions (postpartum depression)	*Postpartum depression*: Edinburgh Postnatal Depression Scale (EPDS)	Repeated Measures ANOVA; Pearson’s Correlation		Results of EPDS were unclear. Pearson’s correlation found that scores on the EPDS Hb and MCV.
Murray-Kolb and Beard, 2007 [2]	Cognition	*IQ*: Shipley Institute of Living Scale (20), Cognitive Abilities test (CAT) measuring 3 domains. *Attention*: Reaction Time Task. *Memory*: Reaction Time task (short-term memory (STM)), Probe Recall Task (recognition), Sternberg Memory Search Task (STM, retrieval and executive functioning). *Learning*: CAT	ANCOVA, assessed differences between groups with IQ as a covariate; repeated-measures ANOVA, assessed change in cognitive variables over time	Iron-treated participants were found to have significantly improved iron status. Significant increases occurred in ID iron treatment and IDA iron treatment groups for ferritin (*p* < 0.001 and *p* < 0.01, respectively) and body iron (*p* < 0.0001 and *p* < 0.01, respectively) The IDA iron-treated participants also had a significant increase in Hb (*p* < 0.0001), haematocrit (*p* < 0.001) and transferrin saturation (*p* = 0.018).	**Cross-sectional baseline comparisons:**
					*Attention:* Composite score significantly lower in IDA group, between iron sufficient and ID (*p* = 0.008; *p* = 0.003, respectively). Performance in attention significantly better than IDA group, in control group and iron-deficient groups (*p* = 0.047 and 0.008, respectively). *Memory*: Composite scores show that iron-sufficient and ID groups scored equally well and significantly better than IDA (*p* < 0.001). Performance in this domain showed the same pattern. *Learning*: Composite score on the learning domain showed that iron-sufficient group scored better than ID group, and there was a significant difference between iron-sufficient and IDA group (*p* = 0.013) and ID and IDA group (*p* = 0.042).
					**Longitudinal analysis:**
					Significant improvement in serum ferritin (*n* = 66) was related to improvements in the attention and learning and memory domains, 5-/7-times, respectively, in women that were non-responders (*n* = 66) (*p* < 0.001). No significant correlation between the size of ferritin change and the cognitive improvement. Women who had significant change in Hb concentration (*n* = 33) completed the attention and memory tasks significantly faster than Hb non-responders (*n* = 80) (*p* < 0.001).
McClung *et al.*, 2009 [16]	Mood and physical performance	*General mood*: Profile of Mood State (POMS)	Two-way repeated measures ANOVA, treatment and time effects	Participation in 8-wk battle combat training had effects on iron status, as shown by elevation (*p* < 0.05) of Hb, red blood cell distribution width and soluble transferrin receptor (sTfR) in both placebo and iron-treated groups. Serum ferritin was diminished (*p* < 0.05) in placebo, but not iron-treated groups.	Group x Time interaction for vigour on the POMS: Iron supplementation had a significant effect on vigour, after Battle CombatTraining (BCT). After stratification by iron status at the beginning of BCT, the positive effects of time on mood stayed significant (*p* < 0.05) for all indicators, except anger, in the iron-deficient group
Verdon *et al.*, 2003 [24]	Depression, anxiety and fatigue	*Depressive and anxiety symptoms*: Questionnaire bref d’auto-évaluation des dimensions dépressive, asthénique et anxieuse	Intention-to-treat protocol χ^2^ and linear regression	Unclear	The iron group showed the largest decrease in cumulative −4.6 (7.5) score for fatigue (−7.5 (8.0)), a difference of 3.0 points, 0.3 to 5.6, *p* = 0.03. Scores for depression were not significant between groups. Depression was not associated with any markers of iron status
Lambert *et al.*, 2002 [23]	Cognition	*Verbal Working Memory, free recall*: Hopkins Verbal Learning Test. *Attention:* Stroop Task. *Processing speed and attention*: Visual Search. *Working memory*: Reading Span Task.	Multiple regression analysis	Ferritin level was increased for both placebo (*t* (58) = 3.73, *p* < 0.001) and iron group (*t* (56) = 9.16, *p* < 0.001). It was found that the extent of increase was larger in the iron group. A significant decrease in Hb concentration was found in the placebo group pre-/post-treatment (*t* (56) = 4.09, *p* < 0.001), and there was no change in Hb level for iron groups pre-/post-treatment (*t* (56) = 1.41, not significant).	*Hopkins Verbal Learning Test*: For the iron group, significant improvement in recall of words from baseline to post-treatment (*t* (56) = 2.40, *p* = 0.01) in the second half list, but no significant relationship between iron treatment and the first half of the list. A significant relationship between Hb change and post-treatment performance on recall for the iron treatment group, second half of the list only (*p* < 0.004). *Reading Span Test*: Multiple regression found a significant relationship between change in serum ferritin and reading span (*p* < 0.01). *Stroop Task*: No relationship found between performance on this task with changed iron status or supplementation. *Visual Search Task*. No treatment x testing interaction found.
Devaki *et al.*, 2009 [20]	Cognition and Emotion (mood)	STM: recall a list of 6 digit numbers, after interpolated activity, asked to recall (not as in Wechsler Intelligence Scale (WAIS)). Long-term memory (LTM): recited the stimulus number, presented four times and then asked to recall one h of interpolated activity (not as in WAIS). IQ: Ravens Progressive Matrices (RPM). WAIS. *Affective behaviour*: Emotional Quotient.	2 × 2 ANOVA with duration as one factor and study group as another	Iron-sufficient, supplement group, ID and IDA groups showed significant increases in Hb, transferrin saturation (TS) and serum ferritin 8 months post-treatment (*p* < 0.01). Iron-sufficient, placebo group showed no increase in iron status.	Iron supplementation groups showed improvements in mean scores of STM, LTM WAIS and RPM, at four- and eight-month follow up, which also reflected changes in haematological parameters. Changes in cognitive scores were much higher in the iron-deficient and iron-deficient anaemic group, compared to iron-sufficient, supplement group. No significant change in Emotion Quotient (EQ) score was seen for any group.
Vaucher *et al.*, 2012 [22]	Primary Fatigue: Secondary: Mood	Fatigue and depressive symptoms: Current and Past Psychological Scale. Fatigue: Multidimensional Assessment of Fatigue score; self-reported health questionnaire, Global Fatigue Index and Severity.	Intention-to-treat analysis	After 6 weeks of iron treatment, significant effects on markers of iron status were found for iron-supplemented groups. Hb: (3 g/L; *p* = 0.001). Ferritin: (6.8 μg/L; *p* = 0.01). MCV: (1.2 fL; *p* = 0.01). sTfR: (−0.4 mg/L; *p* < 0.001) transferrin saturation (6.6%; *p* < 0.001). Similar effects were seen after Week 12.	Patients receiving iron supplement had a 3.5 point improvement (95% CI) in their fatigue score in current and past psychological scale compared to those in placebo group. Iron treatment had a significant effect on the global fatigue index from the Multidimensional Assessment of Fatigue Scale (*p* = 0.03) and its severity index (*p* = 0.03). Iron was not found to have a significant effect on anxiety or depression scores in this study.
	**2: Zinc**				
**Study**	**Dependent Variable**	**Domains**	**Statistical Analysis**	**Changes in Iron/Zinc Status**	**Findings**
Siwek *et al.*, 2009 [17]	Depression	Depressive Index scores on Clinical Global Impression (CGI), Montgomery-Åsberg Depression Rating Scale (MADRS), Hamilton Depression Rating Scale (HDRS), Beck Depression Inventory (BDI). Remission of depression was defined by a score of “very much” on CGI, plus scores of ≤7 on HDRS, ≤10 on MADRS or ≤9 on BDI.	Linear model, mixed-design ANOVA, with repeated factor being test number, and between factors being treatment, antidepressant and treatment resistance. A non-treatment-resistant group and a treatment-resistant group formed.	Zinc-supplemented groups had a significant increase in serum zinc levels at Weeks 6 and 12. Non-zinc-supplemented groups saw an increase of zinc at Week 12. Treatment-resistant groups demonstrated lower levels of zinc than treatment- and non-treatment-resistant groups.	A significant negative correlation between zinc levels and MADRS scores at Week 12 was found, when either all (*p* < 0.0001) or zinc supplemented patients were taken into the analysis
Nowak *et al.*, 2003 [18]	Depression	Depression Index: HDRS and BDI.	Group differences were assessed using *t*-test and multiple ANOVA with two between-subjects factors (placebo *vs.* zinc treatment)	Unclear	Scores of the HDRS were significantly reduced over time: *F* (3, 28) = 5.091; *p* < 0.001, placebo, and *F* (3, 20) = 29.578 *p* < 0.001, zinc group. The group effect (*F* (1, 48) = 4.275, *p* = 0.049) and time effect (*F* (1, 48) = 21.683; *p* < 0.001) were statistically significant. There was no significant interaction effect. Zinc improved this reduction at Weeks 6 and 12, compared to treatment (*ca*. 55%). Zinc supplementation significantly improved the reduction in BDI scores at Week 12, compared to placebo (by 40%).
Sawada and Yokoi, 2010 [19]	Depression	Somatic symptoms and mood feelings, like anxiety, sensitivity, anger and tension: Profile of Mood State (POMS) and Cornell Medical Index (CMI).	Data were analysed with Wilcoxon’s signed-rank test	Neither intervention showed a significant change in serum ferritin or Hb concentration. Multivitamin (MV) + zinc significantly increased serum zinc concentration, whereas MV alone did not.	Women who took multivitamins with zinc showed a significant decrease in anger-hostility and depression-dejection scores on the POMS.

All five studies assessing the effect of iron supplementation on memory found significant improvements on some aspects of memory regardless of whether the participants had been classified as having ID or IDA at the beginning of the clinical trial. Differences in dose ranged between 100 mg elemental iron once per day [14] to 130 mg elemental iron twice daily [7]. Further information on dosage can be found in Table 1. Murray-Kolb and Beard [2] administered a supplement including 160 mg ferrous sulphate with 60 mg of elemental iron once daily. Four studies found improvement on tasks of working memory after treatment with iron supplementation [2,15,21,23]. Additionally, Devaki *et al.* [20] reported a significant improvement on tasks of STM and LTM after iron intervention.

Both Bruner *et al.* [15] and Lambert *et al.* [23] reported significant improvements on verbal working memory post-iron treatment, even in participants with ID. Murray-Kolb and Beard [2] found no significant differences between iron ID participants and iron-sufficient controls on measures of memory; however, participants who were serum ferritin responders did significantly improve on these tasks.

Lambert *et al.* [23] also reported a relationship between changes in Hb concentration and improvement in recall of recently heard words, changes in serum ferritin and improvement in reading span task and post-treatment ferritin level and post-treatment reading-span performance. Similarly, Beard *et al.* [21] reported significant correlations between mean corpuscular volume (MCV) and performance on the digit symbols task at nine months postpartum.

##### Learning

Two studies examined the effect of iron supplementation on learning. One study found an overall effect of learning on the Hopkins Verbal Learning Test (HVLT) scores between treatment and placebo group in groups of females with ID in the absence of anaemia [15]. Bruner *et al.* [15] noted that both iron and placebo groups recalled more words on the HVLT recall task after each successive trial; however, there was no significant difference between these groups. Although they found that both groups did better after intervention, it was noted that girls who received iron supplementation recalled more words on each trial than girls who received the placebo. Murray-Kolb and Beard [2] reported that serum ferritin responders showed significant improvement in their learning abilities. They reported that improvements in learning for ferritin responders were five- to seven-times greater than the improvement in women who did not have a significant increase in serum ferritin.

##### Intellectual Ability

Beard *et al.* [21] found significant improvements on Ravens Progressive Matrices (RPM) scores, for mothers receiving iron supplementation, and they noted that their scores were almost identical to those of control non-anaemic mothers at the end of the treatment period. They also report that anaemic mothers who did not receive iron had no change in cognitive performance over time. Furthermore, they noted a correlation between increased Hb concentration at 10 weeks and improved scores on the RPM. Similarly, Devaki *et al.* [20] observed that iron supplementation in adolescents with either ID or IDA was associated with significant improvement in intellectual ability in comparison to healthy controls receiving iron or healthy controls with placebo.

#### 3.9.2. Iron and Mood

Studies investigating whether increasing iron status effects changes on parameters of mood were mixed, and those presented within this literature review should be interpreted with caution. Two studies found no association between changes in iron status and depressive symptoms or other mood aspects, including anxiety [22,24]. McClung *et al.* [16] reported that battle combat training resulted in significant improvement in mood state over time on all subscales of the Profile of Mood State (POMS) and the total score for both groups. Despite this, the only subscale in which there was a significant group by time interaction was for vigour, indicating that this was the only subscale on which iron supplementation had any effect [16]. Alternatively, Beard *et al.* [21] reported finding a 25% improvement in previously iron-deficient mothers’ depressive scale following an iron supplement compared with a placebo-controlled group. Multivariate analysis suggested a strong link between the Edinburgh Post-Natal Depression Scale (EPNDS) and markers of iron status, specifically mean corpuscular volume and Hb concentration [21].

#### 3.9.3. Zinc and Depression

Two studies assessed the efficacy of zinc supplementation as an adjunct to antidepressant treatment for depression. Nowak *et al.* [18] found statistically significant group effects (treatment* vs.* placebo) and time effects (pre-treatment* vs.* post treatment) for zinc supplementation as an adjunct to antidepressant therapy. It was observed that zinc supplementation significantly augmented the reduction in Hamilton Depression Rating Scale score at Week 12 of treatment [18]. Similarly, Siwek *et al.* [17] found the zinc supplementation as an adjunct to tricyclic antidepressant treatment significantly reduced depressive symptoms measured by the Montgomery–Åsberg depression rating scale (MDRAS). They reported this result in participants who were deemed treatment resistant. The same pattern was not observed in depressed individuals who were treatment responsive.

Only one study was found that examined the effect of zinc supplementation on mood states. Sawada and Yokoi [19] discovered that individuals who were provided with multivitamin (A, D, B1, B2, B6, B12, niacin and folic acid) supplements containing 7 mg added of elemental zinc significantly decreased scores on anger-hostility and depression-dejection scales on the POMS (*p* < 0.05).

## 4. Discussion

The current systematic literature review found five studies that suggested reduced iron status, regardless of whether it is ID or IDA, has a detrimental impact on cognitive function in pre-menopausal women. All five studies found that increasing iron through supplementation had a positive influence on the performance on tasks measuring certain aspects of memory. Four of these studies found significant improvements on tasks of working memory [2,15,21,23], while one noted improvements on tasks of STM and LTM [20]. Only one of the five studies examining the effects of increasing iron status on cognitive functioning found a positive effect on attention [2]. Furthermore, some improvement was found in learning ability in women who were serum ferritin responders, but again, only in one study [2]. In addition, two studies found significant improvements in intellectual ability [20,21]. The results of the current literature review suggest that some, but not all, aspects of cognitive function are impacted by lowered iron status. Memory, particularly working memory and intellectual ability, may be affected by states of iron insufficiency and are potentially improved as iron status is improved. Furthermore, conclusions about the influence of low iron on attention and learning in premenopausal women cannot be made based on this review alone, and further research is required to fully understand these results [2]. These findings are somewhat in contrast to those reported by a meta-analysis on a similar topic. Falkingham *et al.* [26] reported no evidence to suggest any overall effect on memory, psychomotor skills or scholastic achievement in either older children or pre-menopausal women, but did find effects of iron supplementation on aspects of attention and intelligence. In a recent review, Murray-Kolb [27] discussed a number of intervention studies on pre-menopausal women and reported on five that found improvements on differing measures of memory after iron supplementation. Furthermore, Murray-Kolb discussed a number of observational studies and reported a correlation between memory, attention, learning and spatial ability. There is still a relative paucity of research in this area highlighting the need for carefully-designed, randomized controlled trials.

The effect of iron supplementation and its role in improving mood states is not clear, and the interpretation of studies to date is hindered by the range of instruments used to measure mood. Two of the four studies that looked at the effect of increasing iron on mood found no significant improvement of depressive symptoms after iron supplementation, despite a significant increase in iron status [22,24]. However, these studies were only exploring the effect of iron supplementation on depressive symptoms as a secondary outcome, and the inclusion of more comprehensive depressive scales may have yielded different results. On the other hand, a number of previous studies have observed a relationship between low iron status and mood, indicating a potential role for iron in the development of mild depressive symptoms [28,29]. The study by Beard *et al.* [21] reported a significant improvement in depressive symptoms after iron supplementation. The participants of this study did not have a diagnosis of MDD, and the researchers were looking specifically at the risks of ID in developing postpartum depression. Nonetheless, the results of this study appear to indicate that women with IDA may be susceptible to developing postpartum depression and that treatment with iron can reverse these symptoms. McClung *et al.* [16] found a significant positive effect over time of iron supplementation on scores on the POMS for the subscale vigour, but failed to find any similar effect for the depression subscale. While results are mixed, previous research suggests that there is a relationship between low iron status and mood. For example, Vahdat Shariatpanaahi *et al.* [28] report finding that serum ferritin levels were significantly lower (11 μg/L) in individuals with depression in comparison to healthy controls. Furthermore, they reported that the incidence of ID was higher in depressed individuals by 15%. Similarly, a study on postpartum depression discovered a relationship between lowered Hb concentration and depressive symptoms in comparison to mothers with a normal Hb concentration postpartum [29]. This area of research warrants well-designed randomized controlled trials with a large population sample utilizing standardized and valid measures of depressive symptoms.

Clinical intervention trials looking at the effect of zinc supplementation on aspects of mood are limited, and no intervention trials looking at the effect of zinc supplementation on improving cognition were found using the current search criteria. Results for zinc studies found in this review are similar to that of Lai *et al.* [30], as little research in this area has been conducted since then. Zinc has been found to be effective in the treatment of depressive symptoms, both as an adjunct to traditional antidepressant medication and as a stand-alone treatment. The findings by Sawada and Yokoi [19] are preliminary and need to be interpreted with caution; however, they do suggest a role for zinc in the treatment of mild depressive symptoms in the absence of a confounding antidepressant treatment. That being said, both the treatment and placebo groups were provided with additional multivitamins, so intervention studies using only zinc as a treatment are required to clearly elucidate the role of zinc in the treatment of depression.

While no studies were found looking at the association between low zinc status and cognitive function, there is evidence to suggest that there is a potential role for zinc in improving aspects of cognitive ability [31,32,33]. For example, Keller, Chu and Coffield [31] observed that pregnant women with zinc deficiency performed significantly worse on the Raven’s Progressive Matrices than those without zinc deficiency. Furthermore, Maylor *et al.* [34] in a randomized controlled trial on cognitive function in healthy middle-aged and older adults (>70) found a significant positive effect of zinc supplementation on spatial working memory, but a detrimental effect on one measure of attention. As with all of the variables included within this review, further well-designed randomized controlled trials in adults are warranted to better understand the causal relationship between zinc and cognition.

Heterogeneity among the included studies with regard to population and outcome measures used make it difficult to compare the results completely. Only one study controlled for the potential effect of inflammation, as measured by C-reactive protein, on iron status in participants [2]. This is a useful variable to control for, as inflammation caused by chronic disease (e.g., tuberculosis or endocarditis), autoimmune disease (e.g., Crohn’s disease), obesity or exercise can negatively impact iron status and in itself can result in ID [35]. Furthermore, Murray-Kolb and Beard [2] were the only researchers to use IQ as a covariate when looking at learning ability and intellectual functioning. Despite this, most studies matched their participants for socioeconomic status, education, age and physical activity, and most of the studies had procedures to monitor compliance. Generally, all studies were at low risk of selection, performance, detection and attrition biases, as randomization or allocation procedures were made explicit; blinding after allocation was explicit and, for the most part, attrition rates were reported. However, it should be noted that the study by Devaki *et al.* [20] is at risk of selection, performance and detection biases, because randomization of participants did not occur, nor were the investigators or subjects blinded to the group in which they were. While all studies included control groups, Bruner *et al.* [15] split their ID participants into control or treatment groups and, thus, were unable to measure baseline differences between normal status and iron-deficient adolescents. Devaki *et al.* [20] had a supplemented control group; however, they neglected to have iron-deficient or anaemic participants take a placebo. Therefore, their study was not truly placebo controlled.

The current search yielded no results looking at increasing zinc and its effects on cognitive functioning and limited results looking at zinc supplementation and its effects on depression. Secondly, no studies were found that used dietary methods to increase iron or zinc, and lastly, there was limited literature looking specifically at women of childbearing age. As a consequence, the search terms for this review were broadened to include oral iron supplementation, as well as male participants. This means that the results of the current review cannot be generalized specifically to pre-menopausal women, nor can it comment on the effectiveness of increasing dietary iron and zinc on improved mood or cognitive functioning, which was the original intended purpose of this review. Thus, studies investigating the relationship between dietary iron and zinc on markers of mood and cognition, and whole of diet interventions aimed at improving neuropsychological functioning are required.

## 5. Conclusions

The current review has explored the effect of increasing elemental iron, or zinc, on aspects of mood and cognition. Some evidence has been found to suggest a positive effect of increasing iron status on cognitive functioning. It also seems that this positive effect occurs regardless of whether the participant was initially iron insufficient or iron deficient with anaemia. Results are mixed as to whether iron deficiency impacts on general mood; however, further research in this area is warranted. The current review also found some evidence to suggest that increasing zinc may have a positive impact on mood. Further study in the area, utilizing well-designed randomized controlled designs, is needed in order to strengthen the evidence suggesting a causal relationship. Given the risk posed for women of child bearing age for iron and zinc deficiencies, it would be useful to have further research specific to this population group. Furthermore, research exploring the effectiveness of using dietary approaches to increasing iron and zinc in pre-menopausal women to improve cognitive functioning and mood states is warranted.

## Appendix

### Appendix 1. Initial Search Strategies

From Proquest Health and Medical complete search.

Iron/mood/cognition: (ab((iron) AND (deficien* OR status OR intake OR dietary OR nutrition*)) AND peer(yes)) AND ((su((mood) OR (depress*) OR (affect*)) AND peer(yes)) OR (cognit* AND peer(yes))) AND (su((wom?n) OR (“female*”)) AND peer(yes))

Zinc/mood/cognition: ((all(“zinc deficien*” OR “low zinc” OR “zinc store*” OR “zinc status” OR “decree* zinc” OR “deplete* zinc” OR “dietary intake of zinc”) AND peer(yes)) AND (ab(mood OR affect* OR depress*) AND peer(yes))) OR ((all(“zinc deficien*” OR “low zinc” OR “zinc store*” OR “zinc status” OR “decree* zinc” OR “deplete* zinc” OR “dietary intake of zinc”) AND peer(yes)) AND (ab(Cognit*) AND peer(yes)))

### Appendix 2. Refined Search Strategies

From Proquest Health and Medical Complete search.

Iron/mood/cognition/women: (ab((iron) AND (deficien* OR status OR intake OR dietary OR nutrition*)) AND peer(yes)) AND ((su((mood) OR (depress*) OR (affect*)) AND peer(yes)) OR (cognit* AND peer(yes))) AND (su((wom?n) OR (“female*”)) AND peer(yes))

Zinc/mood/cognition/women: ((zinc) AND (deficien* OR status OR intake OR dietary)) AND (su((mood) OR (depress*) OR (affect*)) OR cognit*) AND su((wom?n) OR (“female*”))
